# Fuzzy adaptive fault-tolerant control for an unmanned surface vehicle with prescribed tracking performance

**DOI:** 10.3389/frobt.2025.1576171

**Published:** 2025-04-09

**Authors:** Yunuo Bao, Ji Gao, Peng Peng

**Affiliations:** ^1^ School of Public Affairs, Nanjing University of Science and Technology, Nanjing, China; ^2^ 704 Research Institute, China State Shipbuilding Corporation Limited, Shanghai, China

**Keywords:** unmanned surface vehicles (USVs), actuator faults, trajectory tracking, fault-tolerant control, fuzzy adaptive control

## Abstract

Unmanned surface vehicles (USVs), as a type of marine robotic systems, are widely used in various applications such as maritime surveillance, environmental monitoring, and cargo transportation. This article addresses the trajectory tracking control issue for an USV subject to model uncertainties and actuator faults. A logarithm barrier Lyapunov functions based predefined tracking control scheme is proposed to regulate the position error of the USV into predefined performance region. Then, to ensure the predefined transient and steady state tracking performance of the USV in the presence of actuator faults, we propose an adaptive fuzzy fault-tolerant controller to address the actuator faults. Additionally, to deal with the uncertainties arising from the USV system model, fuzzy logic systems are utilized to estimate the unknown hydrodynamic parameters. Based on the Lyapunov stability criterion, it can be demonstrated that all the closed-loop signals are bounded. Finally, the validity of the developed control scheme is demonstrated from simulation results.

## 1 Introduction

Motivated by their substantial commercial and military importance, unmanned surface vehicles (USVs) have become a focal point in the domains of control theory and engineering (Su et al., 2025b; Shan et al., 2024; Ye et al., 2024; Martinsen et al., 2020; Berman et al. 2020; Wang W. et al., 2024). The commercial worth is manifested in areas such as ocean data acquisition, resource prospecting and construction, as well as bathymetric and environmental assessments (Su et al., 2022b). The military utilizations encompass intelligence gathering, surveillance activities, reconnaissance missions, countermine operations, and submarine hunting (Wen et al., 2022). In general, a dynamic positioning system pertains to the control mechanism of an USV operating in a fully actuated, low-velocity mode (Gao and Li 2024). Its objective is to uphold the USV’s position and orientation at a stationary location or a designated point along its intended path (Gao et al., 2023). The purpose of path-following control is to guide an USV along a prescribed trajectory, often functioning in an underpowered state, navigating it independently through the designated course while maintaining an optimal velocity profile (Wei et al., 2023). Trajectory tracking entails an USV adhering to a precise spatial and temporal path with stringent timing constraints, holding immense importance in marine operations for ensuring safe navigation, minimizing emissions, and conserving energy (Zhang et al., 2024c; Su et al., 2025a). In contrast to path-following, in addition to the steering control algorithm, a defined velocity regulation is now required (Shan et al., 2023).

Currently, a multitude of difficult problems pertain to the control of trajectory tracking for underpowered USVs, with two of these challenges being tackled in this paper. The primary concern revolves around tracking efficacy. Swift and precise trajectory tracking is indispensable for the progression of autonomous capabilities in marine settings and the operational excellence of USVs, particularly in the occurrence of faults (Hu et al., 2016). Relying on the premise of an accurate dynamical representation of the USV, convergence to zero of the tracking deviations was ensured in (Jiang 2002). Accounting for modeling inaccuracies or external disruptions, the boundedness of closed-loop signals was upheld in (Yang et al., 2014; Park et al., 2017). Although adjustable, the predefined values cannot be set for either the convergence rate of the tracking errors or the extent of the residual set, as they are contingent upon uncertain system parameters. The motion control of surface vehicles, particularly focusing on user-specified transient and steady-state performance, was highlighted in (Dai et al., 2016; He et al., 2019; Wang G. et al., 2024). Assurance was given that the tracking errors would enter a predetermined zone at a specified speed. However, because of the exponential convergence performance, the closed-loop errors ultimately approach and settle within the residual set over an indefinite period. In practice, precise trajectory tracking must invariably be accomplished within a specific time and resist the occurrence of failures (Zhang and Yang 2020a). Fortunately, the fault-tolerant prescribed performance control is an effective method. The authors in (Zhang and Yang 2020b) firstly develop a new fault-tolerant prescribed tracking control method for unknown Euler–Lagrange systems, where a novel fault compensation strategy is proposed to ensure the prescribed tracking accuracy and time even if facing the actuator failures. In Zhang et al. (2024a), a challenging fault-tolerant prescribed performance control problem is solved for wheeled mobile robots by designing a novel mixed-gain adaption technology. The work in Wu et al. (2024) develops a novel finite-time prescribed performance control for stochastic systems subject to actuator faults.

As the complexity of modern USVs continues to rise, faults have become virtually unavoidable, potentially causing a decline in performance, system instability, or, in the worst-case scenario, catastrophic accidents (Hao et al., 2021; Andreotti et al., 2024; Wu et al., 2025). Among all potential fault types, actuator faults pose a particularly grave threat, as they can directly alter the behavior of the system through erroneous actuator actions (Li 2019). Motivated by these observations, a fault-tolerant trajectory tracking for an USV to counteract actuator faults was developed in (Wan et al., 2022). The authors in Liu et al. (2024) proposed a leader-following fault-tolerant tracking control method for multiple USVs. In Li et al. (2024), the problem of fuzzy adaptive tracking control for USVs subject to actuator faults has been addressed. To realize the predefined transient performance tracking, an event-based intelligent fault-tolerant control approach was developed in (Su et al., 2022a).

Driven by these observations, this article investigates the adaptive predefined performance trajectory tracking control issue for an USV in the presence of actuator faults. The main contributions of the article are summarized as follows: (1) By fusing the fuzzy logic system into adaptive mechanism, an adaptive fuzzy tracking strategy is developed for an uncertain USV system. The proposed control laws for kinematics and kinetics of the USV can accommodate significant model uncertainties. In this article, we dispense with the assumption concerning the availability of precise or partial information on the dynamic model parameters of the USV; (2) by designing the intermediate control laws in surge and yaw in the kinetic layer design, an adaptive fault-tolerant controller is proposed to address actuator faults and create controllers that do not rely on any prior information about the unknown system parameters or actuator malfunctions; (3) a position error constraint mechanism is employed to solve the underactuation of the USV. By managing the shifted tracking variable, the proposed method allows the user to predetermine both the convergence time and the control precision.

## 2 System description and preliminaries

### 2.1 System model

From Xu et al. (2024b), the USV model with kinematics and kinetics is given by

$$\begin{array}{cc} \dot{x}= & u\cos\psi -v\sin\psi \\ \dot{y}= & u\sin\psi +v\cos\psi \\ \dot{\psi }= & r\\ \dot{u}= & \frac{1}{M_{1}}\left(M_{2}vr-D_{1}u+T_{u}+H_{u}\right)\\ \dot{v}= & \frac{1}{M_{2}}\left(-M_{1}ur-D_{2}v+H_{v}\right)\\ \dot{r}= & \frac{1}{M_{3}}\left(\left(M_{1}-M_{2}\right)uv-D_{3}r+T_{r}+H_{r}\right) \end{array}$$ (1)

where x and y are the displacement in surge and sway, and ψ is the yaw angel; u and v are the linear speeds in surge and sway, and r is the yaw rate; $M_{1}$ , $M_{2}$ , and $M_{3}$ represent the USV’s inertia; $D_{1}$ , $D_{2}$ , and $D_{3}$ denote the damping terms; $T_{u}$ and $T_{r}$ are the surge force and yaw moment; $H_{u}$ , $H_{v}$ , and $H_{r}$ are the external disturbances.

### 2.2 Actuator faults

In practice, it is preferable for the controlled USV to possess fault resistance. The paper considers the following actuator failures as

$$T_{u}=\lambda_{u}Q_{u}+p_{u},T_{r}=\lambda_{r}Q_{r}+p_{r}$$ (2)

where $Q_{u}$ and $Q_{r}$ denote the designed control laws, acting as the actuator inputs; $\lambda_{u}$ and $\lambda_{r}$ are the multiplicative faults; $p_{u}$ and $p_{r}$ are the additive faults. When $\lambda_{u}=\lambda_{r}=1$ and $p_{u}=p_{r}=0$ , the USV’s actuators are fault-free.

### 2.3 Problem statement

The article concentrates on the trajectory tracking control issue for the USV in a desired reference $\left(x_{r},y_{r}\right)$ . We define the following coordinate transformations as

$$e_{1}=\sqrt{z_{1}^{2}+z_{2}^{2}},e_{2}=\ln\left(\frac{\eta_{\psi }+\sigma_{1}}{\eta_{\psi }-\sigma_{1}}\right),e_{3}=u_{a}-u,e_{4}=r_{a}-r$$ (3)

where $e_{1}$ is the position error; $z_{1}=x_{r}-x$ and $z_{2}=y_{r}-y$ are surge and sway displacement errors; $e_{2}$ is a barrier function to manage the control coefficient in sway $\sigma_{1}=\frac{1}{e_{1}}\left(z_{1}\sin\psi -z_{2}\cos\psi \right)$ , and $\eta_{\psi }=\sqrt{1-w^{2}}$ is a design parameter with w>0 ; $e_{3}$ and $e_{4}$ are the surge and yaw speed errors.

To continue, we introduce the following assumptions.

Assumption 1. The unknown constants $\lambda_{u}$ , $\lambda_{r}$ , $p_{u}$ , and $p_{r}$ are bounded. Then, there are unknown constants $\lambda_{u,\inf}$ , $\lambda_{r,\inf}$ , $\lambda_{u,\sup}$ , $\lambda_{r,\sup}$ , $p_{u,\sup}$ , $p_{r,\sup}$ satisfying $0<\lambda_{u,\inf}\leq \lambda_{u}\leq \lambda_{u,\sup}<1$ , $0<\lambda_{r,\inf}\leq \lambda_{r}\leq \lambda_{r,\sup}<1$ , $|p_{u}|\leq p_{u,\sup}$ , and $|p_{r}|\leq p_{r,\sup}$ .

Assumption 2. The given reference trajectory $\left(x_{r},y_{r}\right)$ and its first and second derivative are bounded.

Assumption 3. Zhang et al. (2024c) The sway speed v is passive-bounded.

Assumption 4. The disturbance terms $H_{u}$ , $H_{v}$ , and $H_{r}$ are bounded.

## 3 Tracking control design

### 3.1 Kinematics design

Based on Equation 1, and differentiating $e_{1}$ in Equation 3, we have

$${\dot{e}}_{1}=\sigma_{1}v-\sigma_{2}u+U_{1}$$ (4)

where $\sigma_{2}=\frac{1}{e_{1}}\left(z_{1}\cos\psi +z_{2}\sin\psi \right)$ and $U_{1}=\frac{1}{e_{1}}\left(z_{1}{\dot{x}}_{r}+z_{2}{\dot{y}}_{r}\right)$ . For the sake of ensuring the controllability of the dynamics ${\dot{e}}_{1}=\sigma_{1}v-\sigma_{2}u+U_{1}$ Equation 4, we attempt to constrain $\sigma_{2}$ such that $|\sigma_{2}|>w>0$ , where 0<w<1 is a design parameter. Note from the definition of $\sigma_{1}$ and $\sigma_{2}$ that $\sigma_{1}^{2}+\sigma_{2}^{2}=1$ . Thus, $|\sigma_{2}|>w$ can be ensured by constraining $|\sigma_{1}|<\eta_{\psi }$ . Then, the variable $e_{2}$ in the form of logarithm-type barrier function is used.

Based on the prearranged time T>0 and the prescribed accuracy $\mu_{2}>0$ , we employ the constraint function in Zhang et al. (2024c) described as

$$\vartheta \left(t\right)=\mu_{1}\iota^{g}\left(t,T\right)+\mu_{2}$$ (5)

with

$$\iota \left(t,T\right)=\left\{\begin{array}{cc} 0.5\cos\left(\frac{\pi t}{T}\right)+0.5, & t<T\;\\ 0, & t\geq T\; \end{array}\right.$$ (6)

in which the design parameter $\mu_{2}>0$ is the specified steady-state control accuracy; $\mu_{1}\geq 0$ is a constant and should meet $\mu_{1}+\mu_{2}>e_{1}\left(0\right)$ ; $g\in N^{+}$ are the designed constants.Subsequently, utilizing the aforementioned constraint mechanism for the position error, the shifted error is defined as

$${\bar{e}}_{1}=\ln\left(\frac{e_{1}}{\vartheta -e_{1}}\right).$$ (7)

Then, we employed the following Lyapunov function to manage the shifted position error

$$V_{1}=\frac{1}{2}{\bar{e}}_{1}^{2}.$$ (8)

Based on Equation 4, the time derivative of $V_{1}$ in Equation 8 is

$${\dot{V}}_{1}={\bar{e}}_{1}\eta_{p}\left(\sigma_{1}v-\sigma_{2}u+U_{1}-U_{3}\right)$$ (9)

where $\eta_{p}=\frac{\vartheta }{e_{1}\left(\vartheta -e_{1}\right)}$ and $U_{3}=\frac{e_{1}\dot{\vartheta }}{\vartheta }$ .

Next, the virtual surge speed signal can be proposed as

$$u_{a}=\frac{1}{\sigma_{2}}\left(l_{1}{\bar{e}}_{1}\eta_{p}+\sigma_{1}v+U_{1}-U_{3}\right)$$ (10)

where $l_{1}>0$ is a design parameter.

By invoking Equation 10 into Equation 9, one has

$${\dot{V}}_{1}=-l_{1}\eta_{p}^{2}{\bar{e}}_{1}^{2}+\sigma_{2}\eta_{p}{\bar{e}}_{1}e_{3}.$$ (11)

By differentiating $e_{2}$ in Equation 3, we get

$${\dot{e}}_{2}=\frac{\phi_{\psi }}{2\eta_{\psi }}\left(\sigma_{2}r+\frac{1}{e_{1}}\left(u\sigma_{1}\sigma_{2}+v\sigma_{2}^{2}\right)+U_{2}\right)$$ (12)

where $\phi_{\psi }=\frac{1}{\eta_{\psi }^{2}-\sigma_{1}^{2}}$ and $U_{2}=U_{1}-U_{3}-\frac{1}{e_{1}}\sigma_{1}U_{1}$ .

Define the following Lyapunov function as

$$V_{2}=\frac{1}{4\eta_{\psi }}e_{2}^{2}.$$ (13)

Based on Equation 12, differentiating $V_{2}$ in Equation 13 gives that

$${\dot{V}}_{2}=\phi_{\psi }e_{2}\left(-\sigma_{2}e_{4}+\sigma_{2}r_{a}+\frac{1}{e_{1}}\left(u\sigma_{1}\sigma_{2}+v\sigma_{2}^{2}\right)+U_{2}\right).$$ (14)

Design the virtual yaw rate control law as

$$r_{a}=-\frac{l_{2}\phi_{\psi }}{\sigma_{2}}e_{2}-\frac{1}{e_{1}}\sigma_{1}u-\frac{1}{e_{1}}v\sigma_{2}-\frac{U_{2}}{\sigma_{2}}$$ (15)

where $l_{2}>0$ is a design parameter.

By substituting Equation 15 into Equation 14, one obtains that

$${\dot{V}}_{2}=-l_{2}\phi_{\psi }^{2}e_{2}^{2}-\phi_{\psi }\sigma_{2}e_{2}e_{4}.$$ (16)

### 3.2 Kinetics design

Due to the unknown fault parameters, we define

$$\begin{array}{cc} & k_{u}=\lambda_{u\inf},q_{u}=\frac{1}{k_{u}},\omega_{u}=p_{u\sup}\\ & k_{r}=\lambda_{r\inf},q_{r}=\frac{1}{k_{r}},\omega_{r}=p_{r\sup} \end{array}$$ (17)

and ${\hat{q}}_{u}$ , ${\hat{q}}_{r}$ , ${\hat{\omega }}_{u}$ , and ${\hat{\omega }}_{r}$ are the estimates of $q_{u}$ , $q_{r}$ , $\omega_{u}$ , and $\omega_{r}$ , respectively. ${\tilde{q}}_{u,r}=q_{u,r}-{\hat{q}}_{u,r}$ and ${\tilde{\omega }}_{u,r}=\omega_{u,r}-{\hat{\omega }}_{u,r}$ .

By differentiating $e_{3}$ and $e_{4}$ in Equation 3, it yields that

$$\begin{array}{cc} {\dot{e}}_{3}= & W_{u}-\frac{1}{M_{1}}\left(M_{2}vr-D_{1}u+T_{u}+{\bar{H}}_{u}\right)\\ {\dot{e}}_{4}= & W_{r}-\frac{1}{M_{3}}\left(\left(M_{1}-M_{2}\right)uv-D_{3}r+T_{r}+{\bar{H}}_{r}\right). \end{array}$$ (18)

where ${\bar{H}}_{u}=H_{u}+\frac{\sigma_{1}}{M_{1}}H_{v}$ nd ${\bar{H}}_{r}=H_{r}-\frac{\sigma_{1}}{e_{1}^{2}M_{1}}H_{u}-\frac{\sigma_{2}H_{v}}{e_{1}^{2}M_{2}}$ are disturbance terms. $W_{u}=\frac{1}{\sigma_{1}^{2}}\left[{\dot{\sigma }}_{1}\sigma_{2}v-\sigma_{1}{\dot{\sigma }}_{2}v+\frac{1}{M_{1}}\sigma_{1}\sigma_{2}\left(-M_{1}ur-D_{2}v\right)\right]+{\left[\frac{1}{\sigma_{2}}\left(l_{1}{\bar{e}}_{1}\eta_{p}+U_{1}-U_{3}\right)\right]}^{\prime }$ and $W_{r}=\frac{1}{e_{1}^{2}}\left[{\dot{\sigma }}_{1}u-\frac{\sigma_{1}}{M_{1}}\left(M_{2}vr-D_{1}u+T_{u}\right)\right]+\frac{v{\dot{\sigma }}_{2}}{e_{1}^{2}}-\frac{\sigma_{1}}{e_{1}^{2}M_{2}}\left(-M_{1}ur-D_{2}v\right)+{\left[-\frac{l_{2}\phi_{\psi }}{\sigma_{2}}e_{2}-\frac{U_{2}}{\sigma_{2}}\right]}^{\prime }$ are residual terms about derivatives of the virtual control laws $u_{a}$ and $r_{a}$ . The symbol ${\left[\cdot \right]}^{\prime }$ is the derivative calculation.

Select the following Lyapunov function as

$$V_{3}=\frac{M_{1}}{2}e_{3}^{2}+\frac{M_{3}}{2}e_{4}^{2}+\frac{1}{2}{\tilde{\Theta }}_{u}^{T}m_{u}^{-1}{\tilde{\Theta }}_{u}+\frac{k_{u}{\tilde{q}}_{u}^{2}}{2s_{u}}+\frac{{\tilde{\omega }}_{u}^{2}}{2\gamma_{u}}+\frac{1}{2}{\tilde{\Theta }}_{r}^{T}m_{r}^{-1}{\tilde{\Theta }}_{r}+\frac{k_{r}{\tilde{q}}_{r}^{2}}{2s_{r}}+\frac{{\tilde{\omega }}_{r}^{2}}{2\gamma_{r}}$$ (19)

where $m_{u,r}\in R^{n\times n}$ are the designed positive definite matrixes; $k_{u,r}$ , $s_{u,r}$ , and $\gamma_{u,r}$ are the positive constants; ${\tilde{\Theta }}_{u,r}=\Theta_{u,r}-{\hat{\Theta }}_{u,r}$ .

From Equations 17, 18 differentiating $V_{3}$ in Equation 19 results in

$$\begin{array}{ll} {\dot{V}}_{3} & =e_{3}\left(F_{u}\left(X_{u}\right)+\lambda_{u}Q_{u}+p_{u}+h_{u}-h_{u}+{\bar{H}}_{u}\right)\\ & \;+e_{4}\left(F_{r}\left(X_{r}\right)+\lambda_{r}Q_{r}+p_{r}+h_{r}-h_{r}+{\bar{H}}_{r}\right)\\ & \;-{\tilde{\Theta }}_{u}^{T}m_{u}^{-1}{\dot{\hat{\Theta }}}_{u}-\frac{k_{u}{\tilde{q}}_{u}{\dot{\hat{q}}}_{u}}{s_{u}}-\frac{{\tilde{\omega }}_{u}{\dot{\hat{\omega }}}_{u}}{\gamma_{u}}-{\tilde{\Theta }}_{r}^{T}m_{r}^{-1}{\dot{\hat{\Theta }}}_{r}-\frac{k_{r}{\tilde{q}}_{r}{\dot{\hat{q}}}_{r}}{s_{r}}-\frac{{\tilde{\omega }}_{r}{\dot{\hat{\omega }}}_{r}}{\gamma_{r}} \end{array}$$ (20)

where $F_{u}\left(X_{u}\right)=\frac{1}{M_{1}}W_{u}-M_{2}vr+D_{1}u$ and $F_{r}\left(X_{r}\right)=\frac{1}{M_{3}}W_{r}-\left(M_{1}-M_{2}\right)uv+D_{3}r$ are unknown nonlinearities since $M_{1,2,3}$ , $D_{1,2,3}$ are the uncertain parameters. Therefore, from Du et al. (2022); Su et al. (2024a), for given parameters $\varepsilon_{u,r}>0$ , there are the fuzzy logic systems such that

$$\begin{array}{cc} F_{u}\left(X_{u}\right)= & \Theta_{u}^{T}o_{u}\left(X_{u}\right)+\epsilon_{u}\left(X_{u}\right),|\epsilon_{u}\left(X_{u}\right)|\leq \varepsilon_{u}\\ F_{r}\left(X_{r}\right)= & \Theta_{r}^{T}o_{r}\left(X_{r}\right)+\epsilon_{r}\left(X_{r}\right),|\epsilon_{r}\left(X_{r}\right)|\leq \varepsilon_{r} \end{array}$$ (21)

where $\Theta_{u,r}\in R^{n}$ are the ideal weights; $o_{u,r}\in R^{n}$ are the fuzzy basic functions; n is the number of fuzzy rules.

Design the following control laws as

$$h_{u}=l_{3}e_{3}+0.5e_{3}+{\hat{\Theta }}_{u}^{T}o_{u}+{\hat{\omega }}_{u}\tanh\left(\frac{e_{3}}{\kappa_{u}}\right)+\sigma_{2}{\bar{e}}_{1}\eta_{p}$$ (22)

$$h_{r}=l_{4}e_{4}+0.5e_{4}+{\hat{\Theta }}_{r}^{T}o_{r}+{\hat{\omega }}_{r}\tanh\left(\frac{e_{4}}{\kappa_{r}}\right)-\phi_{\psi }\sigma_{2}e_{2}$$ (23)

$$Q_{u}=-\frac{e_{3}{\hat{q}}_{u}^{2}h_{u}^{2}}{\sqrt{e_{3}^{2}{\hat{q}}_{u}^{2}h_{u}^{2}+j_{u}}}$$ (24)

$$Q_{r}=-\frac{e_{4}{\hat{q}}_{r}^{2}h_{r}^{2}}{\sqrt{e_{4}^{2}{\hat{q}}_{r}^{2}h_{r}^{2}+j_{r}}}$$ (25)

where $l_{3}$ , $l_{4}$ , $j_{u,r}$ , and $\kappa_{u,r}$ are positive constants to be designed.

Design the following adaptive laws as

$${\dot{\hat{\Theta }}}_{u}=m_{u}\left(o_{u}e_{3}-\xi_{u1}{\hat{\Theta }}_{u}\right)$$ (26)

$${\dot{\hat{\Theta }}}_{r}=m_{r}\left(o_{r}e_{4}-\xi_{r1}{\hat{\Theta }}_{r}\right)$$ (27)

$${\dot{\hat{q}}}_{u}=s_{u}e_{3}h_{u}-\xi_{u2}{\hat{q}}_{u}$$ (28)

$${\dot{\hat{q}}}_{r}=s_{r}e_{4}h_{r}-\xi_{r2}{\hat{q}}_{r}$$ (29)

$${\dot{\hat{\omega }}}_{u}=e_{3}\gamma_{u}\tanh\left(\frac{e_{3}}{\kappa_{u}}\right)-\xi_{u3}{\hat{\omega }}_{u}$$ (30)

$${\dot{\hat{\omega }}}_{r}=e_{4}\gamma_{r}\tanh\left(\frac{e_{4}}{\kappa_{r}}\right)-\xi_{r3}{\hat{\omega }}_{r}$$ (31)

where $\xi_{u1,r1}$ , $\xi_{u2,r2}$ and $\xi_{u3,r3}$ are positive constants to be designed.

### 3.3 Stability analysis

#### 3.3.1 Theorem 1

With the virtual control laws Equations 10, 15, actual control laws (Equations 24, 25), and adaptive laws Equations 26–31, the USV control system (1) under Assumptions 1 and 2 has two properties:

1) The position error can be managed into the prescribed area $\Omega =\left\{e_{1}\in R:e_{1}<\mu_{2}\right\}$ within the predefined time T .

2) All closed-loop signals are bounded.

From Equations 24, 25, and Lemma 5 in (Liang et al., 2021), one can get

$$\begin{array}{cc} e_{3}\lambda_{u}Q_{u}= & -\frac{\lambda_{u}e_{3}^{2}{\hat{q}}_{u}^{2}h_{u}^{2}}{\sqrt{e_{3}^{2}{\hat{q}}_{u}^{2}h_{u}^{2}+j_{u}}}\\ \leq & -\frac{k_{u}e_{3}^{2}{\hat{q}}_{u}^{2}h_{u}^{2}}{\sqrt{e_{3}^{2}{\hat{q}}_{u}^{2}h_{u}^{2}+j_{u}}}\\ \leq & k_{u}\sqrt{j_{u}}-k_{u}e_{3}{\hat{q}}_{u}h_{u}\\ e_{4}\lambda_{r}Q_{r}= & -\frac{\lambda_{r}e_{4}^{2}{\hat{q}}_{r}^{2}h_{r}^{2}}{\sqrt{e_{4}^{2}{\hat{q}}_{r}^{2}h_{r}^{2}+j_{r}}}\\ \leq & -\frac{k_{r}e_{4}^{2}{\hat{q}}_{r}^{2}h_{r}^{2}}{\sqrt{e_{4}^{2}{\hat{q}}_{r}^{2}h_{r}^{2}+j_{r}}}\\ \leq & k_{r}\sqrt{j_{r}}-k_{r}e_{4}{\hat{q}}_{r}h_{r}. \end{array}$$ (32)

Based on the Young’s inequality, one has

$$e_{3}{\bar{H}}_{u}\leq \frac{1}{2}e_{3}^{2}+\frac{1}{2}{\bar{H}}_{u}^{*2}$$ (33)

$$e_{4}{\bar{H}}_{r}\leq \frac{1}{2}e_{4}^{2}+\frac{1}{2}{\bar{H}}_{r}^{*2}$$ (34)

where ${\bar{H}}_{u}^{*}$ and ${\bar{H}}_{r}^{*}$ are bounded due to the bundedness of disturbances.

By invoking Equations 21–25, Eqautions 26–32, and Equations 33, 34 into Equation 20, we have

$$\begin{array}{ll} {\dot{V}}_{3} & \leq -l_{3}e_{3}^{2}-\sigma_{2}{\bar{e}}_{1}\eta_{p}e_{3}+\omega_{u}\left(|e_{3}|-e_{3}\tanh\left(\frac{e_{3}}{\kappa_{u}}\right)\right)+\frac{1}{2}e_{3}^{2}+\frac{1}{2}{\bar{H}}_{u}^{*2}\\ & \;-l_{4}e_{4}^{2}+\phi_{\psi }\sigma_{2}e_{2}e_{4}+\omega_{r}\left(|e_{4}|-e_{4}\tanh\left(\frac{e_{4}}{\kappa_{r}}\right)\right)+\frac{1}{2}e_{4}^{2}+\frac{1}{2}{\bar{H}}_{r}^{*2}\\ & \;+\xi_{u1}{\tilde{\Theta }}_{u}^{T}{\hat{\Theta }}_{u}+\frac{k_{u}\xi_{u2}}{s_{u}}{\tilde{q}}_{u}{\hat{q}}_{u}+\frac{\xi_{u3}}{\gamma_{u}}{\tilde{\omega }}_{u}{\hat{\omega }}_{u}+k_{u}\sqrt{j_{u}}\\ & \;+\xi_{r1}{\tilde{\Theta }}_{r}^{T}{\hat{\Theta }}_{r}+\frac{k_{r}\xi_{r2}}{s_{r}}{\tilde{q}}_{r}{\hat{q}}_{r}+\frac{\xi_{r3}}{\gamma_{r}}{\tilde{\omega }}_{r}{\hat{\omega }}_{r}+k_{r}\sqrt{j_{r}}+0.5\varepsilon_{u}^{2}+0.5\varepsilon_{r}^{2}. \end{array}$$ (35)

Employing the inequality $0\leq |\overset{˘}{\theta }|-\overset{˘}{\theta }\tanh\left(\frac{\overset{˘}{\theta }}{\kappa }\right)\leq 0.2785\kappa \;\left(\kappa >0,\overset{˘}{\theta }\in R\right)$ gives

$$\begin{array}{cc} \omega_{u}\left(|e_{3}|-e_{3}\tanh\left(\frac{e_{3}}{\kappa_{u}}\right)\right)\leq & 0.2785\kappa_{u}\omega_{u}\\ \omega_{r}\left(|e_{4}|-e_{4}\tanh\left(\frac{e_{4}}{\kappa_{r}}\right)\right)\leq & 0.2785\kappa_{r}\omega_{r}. \end{array}$$ (36)

Using the Young’s inequality Su et al. (2024b); Teng et al. (2024a); Xu Y. et al. (2024); Li et al. (2025), we have

$$\begin{array}{cc} \xi_{u1}{\tilde{\Theta }}_{u}^{T}{\hat{\Theta }}_{u}\leq & -\frac{\xi_{u1}}{2}{\tilde{\Theta }}_{u}^{T}{\tilde{\Theta }}_{u}+\frac{\xi_{u1}}{2}\Theta_{u}^{T}\Theta_{u}\\ \xi_{r1}{\tilde{\Theta }}_{r}^{T}{\hat{\Theta }}_{r}\leq & -\frac{\xi_{r1}}{2}{\tilde{\Theta }}_{r}^{T}{\tilde{\Theta }}_{r}+\frac{\xi_{r1}}{2}\Theta_{r}^{T}\Theta_{r}\\ \frac{k_{u}\xi_{u2}}{s_{u}}{\tilde{q}}_{u}{\hat{q}}_{u}\leq & -\frac{k_{u}\xi_{u2}}{2s_{u}}{\tilde{q}}_{u}^{2}+\frac{k_{u}\xi_{u2}}{2s_{u}}q_{u}^{2}\\ \frac{k_{r}\xi_{r2}}{s_{r}}{\tilde{q}}_{r}{\hat{q}}_{r}\leq & -\frac{k_{r}\xi_{r2}}{2s_{r}}{\tilde{q}}_{r}^{2}+\frac{k_{r}\xi_{r2}}{2s_{r}}q_{r}^{2}\\ \frac{\xi_{u3}}{\gamma_{u}}{\tilde{\omega }}_{u}{\hat{\omega }}_{u}\leq & -\frac{\xi_{u3}}{2\gamma_{u}}{\tilde{\omega }}_{u}^{2}+\frac{\xi_{u3}}{2\gamma_{u}}\omega_{u}^{2}\\ \frac{\xi_{r3}}{\gamma_{r}}{\tilde{\omega }}_{r}{\hat{\omega }}_{r}\leq & -\frac{\xi_{r3}}{2\gamma_{r}}{\tilde{\omega }}_{r}^{2}+\frac{\xi_{r3}}{2\gamma_{r}}\omega_{r}^{2}. \end{array}$$ (37)

By substituting Equation 36 and Equation 37 into Equation 35, it follows that

$$\begin{array}{ll} {\dot{V}}_{3} & \leq -l_{3}e_{3}^{2}-\sigma_{2}{\bar{e}}_{1}\eta_{p}e_{3}+0.2785\kappa_{u}\omega_{u}+0.5\varepsilon_{u}^{2}+\frac{1}{2}e_{3}^{2}+\frac{1}{2}{\bar{H}}_{u}^{*2}\;\\ & \;-l_{4}e_{4}^{2}+\phi_{\psi }\sigma_{2}e_{2}e_{4}+0.2785\kappa_{r}\omega_{r}+0.5\varepsilon_{r}^{2}\frac{1}{2}+e_{4}^{2}+\frac{1}{2}{\bar{H}}_{r}^{*2}\\ & \;-\frac{\xi_{u1}}{2}{\tilde{\Theta }}_{u}^{T}{\tilde{\Theta }}_{u}-\frac{k_{u}\xi_{u2}}{2s_{u}}{\tilde{q}}_{u}^{2}-\frac{\xi_{u3}}{2\gamma_{u}}{\tilde{\omega }}_{u}^{2}+k_{u}\sqrt{j_{u}}+\frac{\xi_{u1}}{2}\Theta_{u}^{T}\Theta_{u}\\ & \;+\frac{k_{u}\xi_{u2}}{2s_{u}}q_{u}^{2}+\frac{\xi_{u3}}{2\gamma_{u}}\omega_{u}^{2}-\frac{\xi_{r1}}{2}{\tilde{\Theta }}_{r}^{T}{\tilde{\Theta }}_{r}-\frac{k_{r}\xi_{r2}}{2s_{r}}{\tilde{q}}_{r}^{2}-\frac{\xi_{r3}}{2\gamma_{r}}{\tilde{\omega }}_{r}^{2}\\ & \;+k_{r}\sqrt{j_{r}}+\frac{\xi_{r1}}{2}\Theta_{r}^{T}\Theta_{r}+\frac{k_{r}\xi_{r2}}{2s_{r}}q_{r}^{2}+\frac{\xi_{r3}}{2\gamma_{r}}\omega_{r}^{2}. \end{array}$$ (38)

Design the total Lyapunov function as

$$V=V_{1}+V_{2}+V_{3}.$$ (39)

From Equations 11, 16, 38, the time derivative of V is

$$\begin{array}{ll} \dot{V} & \leq -{\bar{l}}_{1}{\bar{e}}_{1}^{2}-{\bar{l}}_{2}e_{2}^{2}-{\bar{l}}_{3}e_{3}^{2}-{\bar{l}}_{4}e_{4}^{2}-\frac{\xi_{u1}}{2}\|\Theta_{u}{\|}^{2}-\frac{\xi_{r1}}{2}\|\Theta_{r}{\|}^{2}-\frac{k_{u}\xi_{u2}}{2s_{u}}{\tilde{q}}_{u}^{2}\\ & \;-\frac{\xi_{u3}}{2\gamma_{u}}{\tilde{\omega }}_{u}^{2}-\frac{k_{r}\xi_{r2}}{2s_{r}}{\tilde{q}}_{r}^{2}-\frac{\xi_{r3}}{2\gamma_{r}}{\tilde{\omega }}_{r}^{2}+\frac{\xi_{u1}}{2}\Theta_{u}^{T}\Theta_{u}+\frac{k_{u}\xi_{u2}}{2s_{u}}q_{u}^{2}\\ & \;+\frac{\xi_{u3}}{2\gamma_{u}}\omega_{u}^{2}+k_{r}\sqrt{j_{r}}+\frac{\xi_{r1}}{2}\Theta_{r}^{T}\Theta_{r}+\frac{k_{r}\xi_{r2}}{2s_{r}}q_{r}^{2}+\frac{\xi_{r3}}{2\gamma_{r}}\omega_{r}^{2}\\ & \;+0.5\varepsilon_{u}^{2}+0.5\varepsilon_{r}^{2}+\frac{1}{2}{\bar{H}}_{u}^{*2}+\frac{1}{2}{\bar{H}}_{r}^{*2} \end{array}$$ (40)

where ${\bar{l}}_{1}=\frac{16\left(l_{1}-0.5-0.5\sigma_{2}^{2}\right)}{{\left(\mu_{1}+\mu_{2}\right)}^{2}}$ , ${\bar{l}}_{2}=l_{2}-0.5-0.5\sigma_{2}^{2}$ , ${\bar{l}}_{3}=l_{3}-1$ , and ${\bar{l}}_{4}=l_{4}-1$ .

Then, Equation 40 can be further written as

$$\dot{V}\leq -G_{1}V+G_{2}$$ (41)

where $G_{1}=\min\left\{2{\bar{l}}_{1},4\eta_{\psi }{\bar{l}}_{2},2{\bar{l}}_{3}/M_{1},2{\bar{l}}_{4}/M_{3},\xi_{u1}/\lambda_{\max}\left(m_{u}^{-1}\right),\xi_{r1}/\lambda_{\max}\left(m_{r}^{-1}\right),\xi_{u2},\xi_{r2},\xi_{u3},\xi_{r2}\right\}$ and $G_{2}=+\frac{\xi_{u1}}{2}\Theta_{u}^{T}\Theta_{u}+\frac{k_{u}\xi_{u2}}{2s_{u}}q_{u}^{2}+\frac{\xi_{u3}}{2\gamma_{u}}\omega_{u}^{2}+k_{r}\sqrt{j_{r}}+\frac{\xi_{r1}}{2}\Theta_{r}^{T}\Theta_{r}+\frac{k_{r}\xi_{r2}}{2s_{r}}q_{r}^{2}+\frac{\xi_{r3}}{2\gamma_{r}}\omega_{r}^{2}+0.5\varepsilon_{u}^{2}+0.5\varepsilon_{r}^{2}+\frac{1}{2}{\bar{H}}_{u}^{*2}+\frac{1}{2}{\bar{H}}_{r}^{*2}$ .

Integrating both sides of Equation 41, we have

$$\dot{V}\left(t\right)\leq \exp^{-G_{1}T}V\left(0\right)+\frac{G_{2}}{G_{1}}\left(1-\exp^{-G_{1}t}\right)$$ (42)

Therefore, closed-loop signals ${\bar{e}}_{1}$ , $e_{2}$ , $e_{3}$ , $e_{4}$ , $\Theta_{u}$ , $\Theta_{r}$ , $q_{u}$ , $q_{r}$ , $\omega_{u}$ , and $\omega_{r}$ are both bounded.

Because $0<e_{1}\left(0\right)<\mu_{1}+\mu_{2}$ is met, and ϑ(t) smoothly and momtonically reduces from $\mu_{1}+\mu_{2}$ to $\mu_{2}$ on [0,T] . $e_{1}\left(t\right)<\mu_{2}$ on [T,∞] . Then, the position error can be managed into the prescribed area $\Omega =\left\{e_{1}\in R:e_{1}<\mu_{2}\right\}$ within the predefined time T .

## 4 Simulation verification

The validity of presented control method is demonstrated by the numerical simulation.

The system parameters are chosen as $M_{1}=40.76$ , $M_{2}=55.2$ , $M_{3}=2.72$ , $D_{1}=24.33$ , $D_{2}=77.76$ , $D_{3}=9.64$ . The reference trajectory is selected as $\left(x_{r},y_{r}\right)=\left(10\sin\left(0.1t\right),-10\cos\left(0.1t\right)+10\right)$ . The disturbances are set as $H_{u}=3\sin\left(0.1t\right)$ , $H_{v}=2\sin\left(0.5t\right)\cos\left(0.03t\right)$ , and $H_{r}=0.5\cos{\left(0.4t\right)}^{2}$ The initial conditions are chosen as x(0)=0.6 , y(0)=1.5 , ψ(0)=−2.1 , u(0)=0 , v(0)=0 , r(0)=0 .

It is assumed that the actuators in surge and yaw simultaneously undergo multiplicative faults and additive faults at t=30 s, such that

$$\left\{\begin{array}{cc} T_{u}=Q_{u},T_{r}=Q_{r}, & t<30s\;\\ T_{u}=0.5Q_{u}+10,T_{r}=0.5Q_{r}+5.5, & t\geq 30s.\; \end{array}\right.$$ (43)

The adjusting parameters are given by $l_{1}=1$ , $l_{2}=2$ , $l_{3}=10$ , $l_{4}=10$ , $j_{u}=j_{r}=1$ , $\kappa_{u}=\kappa_{r}=1$ , $\xi_{u1}=\xi_{r1}=0.1$ , $\xi_{u2}=\xi_{r2}=0.01$ , and $\xi_{u3}=\xi_{r2}=0.8$ . The control objective is that the position error can be managed into the prescribed area $\Omega =\left\{e_{1}\in R:e_{1}<0.5m\right\}$ within the predefined time 10s.

**FIGURE 1:**
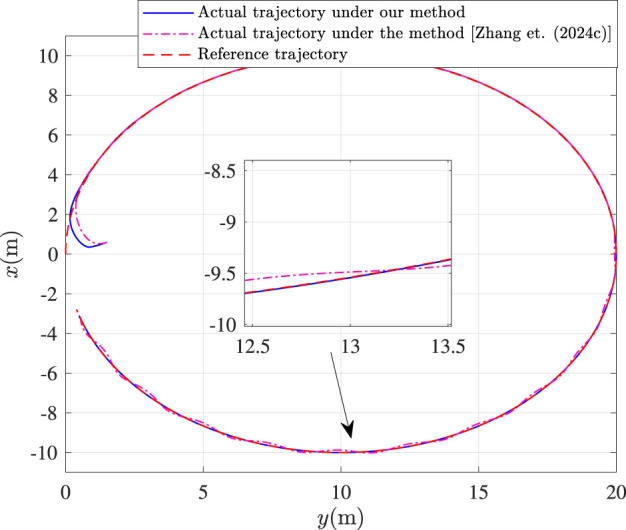
Tracking control performance.

**FIGURE 2:**
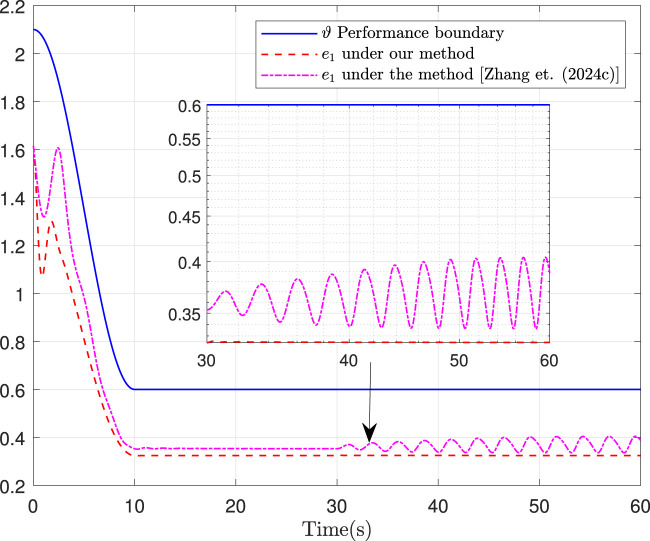
Position error.

**FIGURE 3:**
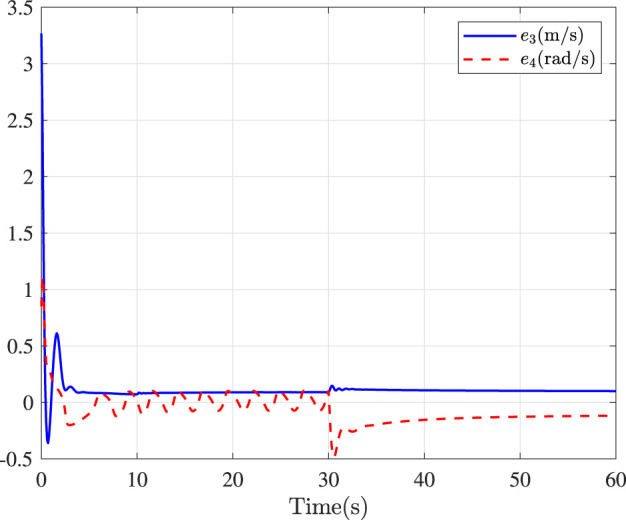
Speed errors in surge and yaw.

**FIGURE 4:**
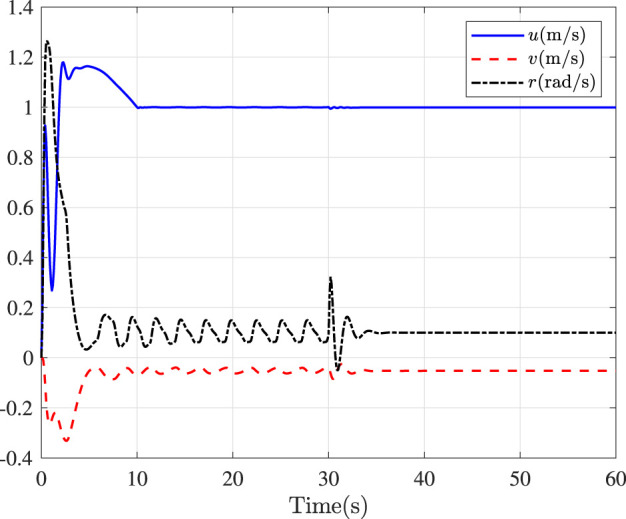
Linear speeds and yaw rate.

**FIGURE 5:**
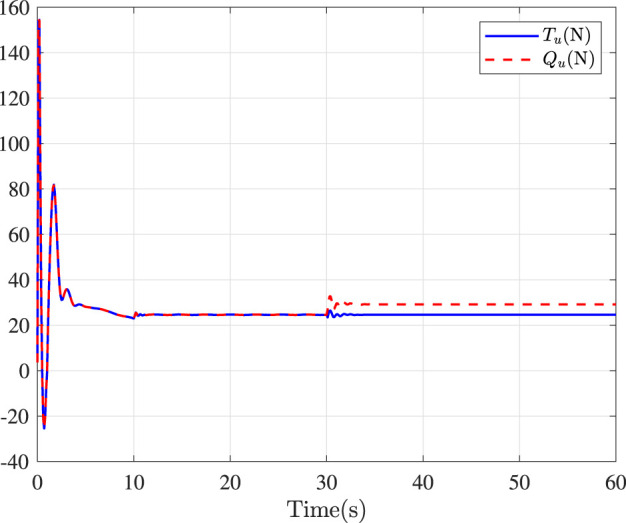
Control inputs in surge.

**FIGURE 6:**
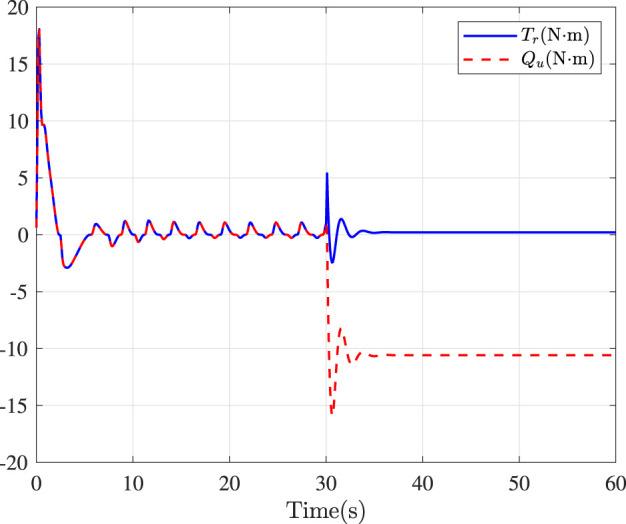
Control inputs in yaw.

**FIGURE 7:**
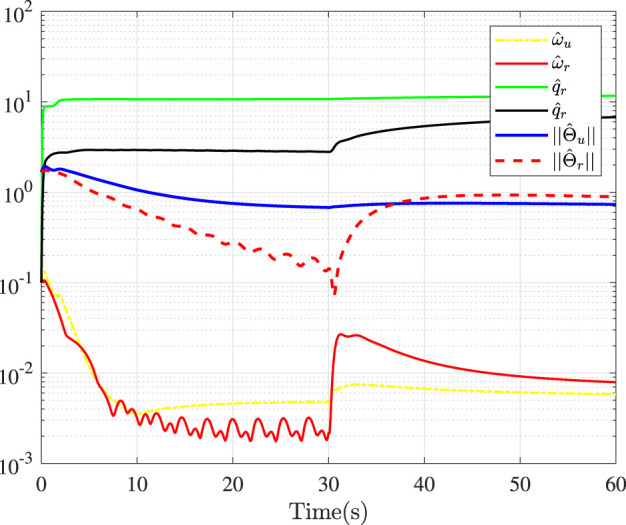
Adaptive parameters.

Fault-tolerance performance: To show the advantage and effectiveness of the proposed fault-tolerant control method, a comparison between the classical prescribed performance tracking control method in (Zhang et al., 2024c) and our method is proposed. In order to ensure the fairness of the comparison, all design parameters are the same. The tracking performance is described in Figure 1, from which it is observable that the control performance is well guaranteed even subject to faults based on our method. Figure 2 describes that the position error can be regulated into the prescribed area $\Omega =\left\{e_{1}\in R:e_{1}<0.5m\right\}$ within the predefined time 10s under our method. Although the performance constraint cannot be violated by using the method (Zhang et al., 2024c), the steady-state tracking accuracy is lower than that our method since actuator failures occur 30 s later. Figure 3 shows that the errors of surge speed and yaw rate approach zero. Figure 4 shows that the speed curves in surge, sway, and yaw. Figures 5, 6 show that the control inputs and outputs of the surge and yaw actuators. The parameter adaptive laws are shown in Figure 7. From Figures 2–7, it can be observed that closed-loop signal are bounded.

**FIGURE 8:**
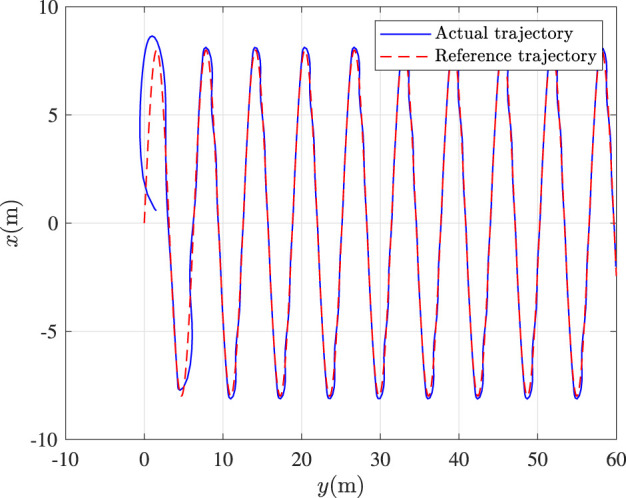
Tracking control performance under actual ocean disturbances.

**FIGURE 9:**
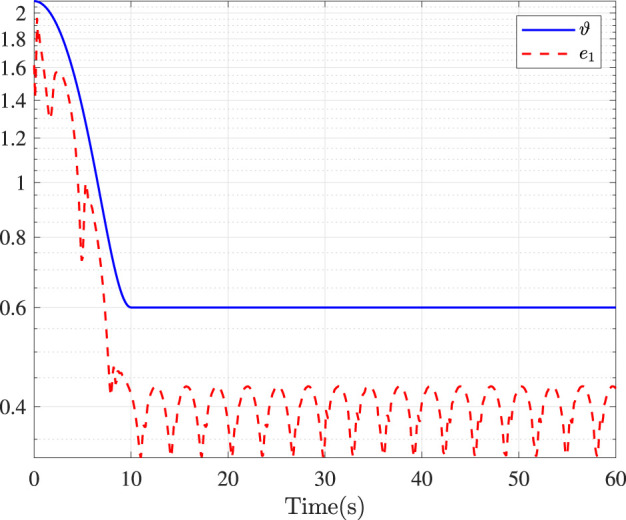
Position error under actual ocean disturbances.

Robustness Test: In order to demonstrate the robustness of the developed control strategy against actual disturbances, the ocean disturbances resulting from waves, winds, and currents are considered from a simulation testing. In simulation, the ocean disturbances are mimicked as a Gaussian random process. Specifically, a second-order bandstop filter is employed to mimic the high-frequency wave motion and a first-order transfer function is used to denote the slow-varying disturbance resulting from wave drift, ocean currents, and winds in the yaw channel. Thus, the disturbance terms are shown as $H_{u}=\sin\left(\psi \right)\bar{y}\left(s\right)$ , $H_{v}=\cos\left(\psi \right)\bar{y}\left(s\right)$ , and $H_{r}=y^{\prime }\left(s\right)$ , where $\bar{y}\left(s\right)$ and $y^{\prime }\left(s\right)$ denote the high-frequency wave motion and the slow-varying environmental disturbances, respectively. For comprehensive details, please see the result in Zhang and Yang (2018). The following trajectory is selected as ${\left[x_{d},y_{d}\right]}^{T}={\left[8\sin\left(t\right),t\right]}^{T}$ . The control parameters in simulation are the same as the previous simulation. Figure 8 describes that the trajectory tracking task is achieved with the presented control method. Figure 9 describes that the position error can be regulated into the prescribed area $\Omega =\left\{e_{1}\in R:e_{1}<0.5m\right\}$ within the predefined time 10s under our method. Since the actual disturbances are considered in this test, the control performance is slightly reduced, which is acceptable.

## 5 Conclusion

This article has investigated the fuzzy adaptive fuzzy predefined performance tracking control issue for the USV subject to actuator faults. By integrating the logarithm barrier Lyapunov functions with adaptive control strategy, the position error is managed into predefined performance region and closed-loop signals are all bounded. Moreover, the proposed adaptive fault-tolerant controller can realize desired control performance even with actuator faults. Future work will focus on the security control problem of the USV with cyber attacks (Teng et al., 2025; Teng et al., 2024b). Considering that all the closed-loop signals are semiglobally uniformly ultimately bounded in this paper, future work also will devote to realize a globally stable result (Zhang et al., 2024b).

## Data Availability

The original contributions presented in the study are included in the article/supplementary material, further inquiries can be directed to the corresponding author.
